# Supplementary material for: Epidemiology, mortality and prevalence of colorectal cancer in ulcerative colitis patients between 2010-2016 in Hungary – a population-based study
Source: PLoS One. 2020 May 14;15(5):e0233238. doi: 10.1371/journal.pone.0233238 (PMC7224530; doi:10.1371/journal.pone.0233238)
Supplement: Attachment — Submitted filename: Response to reviewers Kunovszki et al.docx [file pone.0233238.s002.docx]

Peter Kunovszki

Janssen: Pharmaceutical Companies of Johnson & Johnson

Valérie Pittet, PhD

Academic Editor

PLOS ONE

February 25th, 2020

Dear Ms. Pittet and Reviewers,

we would like to thank the Reviewers for their interest in our work and for the helpful comments and suggestions that will improve the manuscript. We tried to do our best to respond to the points raised.

As indicated below, we have checked all the general and specific comments provided by the Reviewers and Editor(s) and made the necessary changes accordingly to their indications. Original Reviewer comments are in italic face, responses are in regular face.

Yours sincerely,

Peter Kunovszki

***Journal requirements:***

*1. Please ensure that your manuscript meets PLOS ONE's style requirements, including those for file naming.*

We have updated the style of the manuscript, including numbering lines and reformatting figure labels.

*2. We noticed you have some minor occurrence(s) of overlapping text with publication(s).*

We rephrased the above-mentioned parts of the manuscript. However, we made an effort to find the original article from

Saro Gismera C. et al. (1999) (<https://www.ncbi.nlm.nih.gov/pubmed/10231311>),

but since it was written in Spanish, and we don’t speak Spanish, we were unable to find and rephrase the overlapping parts with our manuscript.

*3. In the ethics statement in the manuscript and in the online submission form, please provide additional information about the patient records/samples used in your retrospective study. Specifically, please ensure that you have discussed whether all data/samples were fully anonymized before you accessed them and/or whether the IRB or ethics committee waived the requirement for informed consent. If patients provided informed written consent to have data/samples from their medical records used in research, please include this information.*

We have added the necessary information to both the manuscript and submission form.

The research team had access to anonymized data, the anonymization was performed by the data holder. The re-use of public data (which includes the data used in the study) is guaranteed by law in Hungary (based on Act 63/2012 on the re-use of public data).

The research group had access to the data indirectly, through NHIF according to internal data privacy regulations of NHIF and Regulation (EU) 2016/679 General Data Protection Regulation (GDPR). Due to this, and to the retrospective nature of the study there was no need for patient level consent to the analysis.

*4. Competing interests.*

We would like to modify the competing interests statement to the following due to declarations that I had received after the initial submission was done:

I have read the journal’s policy and the authors of this manuscript have the following competing interests:

PK, JGO, PT, ABo are employees / consultants of Janssen

PLL has been a speaker and/or advisory board member for AbbVie, Arena Pharmaceuticals, Celltrion, Falk Pharma GmbH, Ferring, Genetech, Janssen, Merck, Pharmacosmos, Pfizer, Roche, Shire and Takeda and has received unrestricted research grants from AbbVie, MSD and Pfizer.

TSz has served as advisory board member for AbbVie, EGIS, Pfizer and Takeda, received speaker’s honoraria from Abbvie, Takeda and Ferring and served as part time medical advisor for Hungarian National Health Insurance Fund.

TM received speaker’s honoraria from MSD, AbbVie, Egis, Goodwill Pharma, Takeda, Pfizer and Teva.

KF received speaker’s honoraria from AbbVie, Janssen and Ferring.

ABá: received speaker’s honoraria from Janssen and Ferring.

KSz, ÁM have no conflicts of interest to declare.

This does not alter our adherence to PLOS ONE policies on sharing data and materials.

*5. Data access*

The dataset that was used is held by the National Health Insurance Fund (NHIF) of Hungary (<http://www.neak.gov.hu>, e-mail: [neak@neak.gov.hu](mailto:neak@neak.gov.hu)).

Access to the individual-level data is available after filing a formal data access request to [adatkeres@neak.gov.hu](mailto:adatkeres@neak.gov.hu). Requestors need to accept the terms and conditions of the data request and may need to pay the corresponding data access fee.

The terms of the contract for data access does not allow the reporting of any data of a single individual or results which comes from aggregating the data of less than 10 individuals. Therefore, a de-identified dataset could not be provided. Taking these requirements into consideration, the results can be published.

A supplementary dataset was created which contains the patient counts derived from the original data.

***Reviewers’ comments:***

**Reviewer #1:**

*1. As many may be unfamiliar with your healthcare database it would be helpful to have either a better description or a reference to a description of the database in the manuscript and some highlights as it pertains to this manuscript.*

We revised the manuscript and added some information in the Data collection section about the database used.

*2. Endpoints need to be described better. Primary endpoints are endpoints that your study is powered to detect. Secondary endpoints are those which the study is not powered to detect. Just stating that you had 3 endpoints is not adequate.*

We absolutely agree with the Reviewer that the phrasing „endpoint” is not the most appropriate word we used, as our study is purely descriptive in nature. We decided not to use this terminology at all and rephrased the above-mentioned part of the manuscript and moved these parts to the Methods section.

*3. I would advocate removing figure 2 altogether since you state that you cannot adequately determine the incidence. You do a nice job of explaining why, but that essentially renders most of your data in that table questionable at best. I think keeping he descriptive language is good enough for this.*

Based on the Reviewer’s suggestion, we revised the above-mentioned part of the Manuscript. Figure 2 was removed, and the paragraph has been shortened as well.

*4. On Figure 3, the legend at the top has the male label over the female plot and vv. I would recommend switching that order to make it easier to understand.*

We made the suggested switch in the labelling.

*5. Figure 5 is too difficult to interpret. Please either include the names on the cancers on the Y axis or highlight and name some key cancers within the figure.*

Using some abbreviations, it was possible to include the name of cancers on the Y axis. The caption of the figure was revised accordingly.

*6. Are deaths cancer-specific, disease-specific, or all cause? It seems like they are all cause but I think this needs to be highlighted better. This is also a big weakness of this manuscript because it is hard to know if these cancer patients had more severe disease, or accessed healthcare less and had other comorbidities. This should be discussed more in depth in the discussion.*

We revised and completed the discussion and the limitations parts of the manuscript.

The primary aim of the data collection was not the clinical evaluation of patients, but rather to serve financial and reimbursement purposes. Therefore, no data were available on clinical outcomes, such as laboratory values, disease severity indices, access to healthcare or patient reported outcomes.

*7. Figure 8 is labeled poorly. It seems like it is CRC patients with UC, but from the labeling it appears to be all CRC patients in the country. This labeling should be improved upon.*

We improved the labeling accordingly.

*8. It would be important to know whether the change in mortality from CRC in UC was unique to UC or whether this was similar as to the general population. This data should be collectable using your database and I would recommend including this as well.*

Unfortunately, no information other than the date of birth, date of death and gender could be obtained for the general population in this study; therefore, no further analyses regarding these controls were possible.

We have clarified this circumstance in the Data collection and Discussion parts of the manuscript.

**Reviewer #2:**

*1. Abstract – There is no clear description of the analytical methods used, including type of analysis, matching procedure.*

The study is descriptive in nature. We added this fact to the abstract. We also added some description of the matching procedure to the abstract.

*2. Abstract – The aim in the methods section should be more appropriately located in the background section.*

We made the change proposed, the aims were moved to the Introduction section.

*The manuscript needs to be edited by a thorough English speaker.*

We revised the language used and the manuscript was edited by a thorough English speaker. We hope it matches the journal standard.

*1. The introduction is winding and overly long and could be shortened considerably. What does this study add, why was it done? What gap of knowledge exists and how could this study help`?*

We have shortened the Introduction section by moving some of the content to the Discussion section. The background of the study and the aims of the study are clearly indicated in the revised text.

*2. The aim should be placed in the introduction section, along with a clear hypothesis.*

We moved the aim section to the end of the introduction. Our study is a descriptive, retrospective, epidemiological study; therefore, no formal hypotheses were formulated.

*3. The English is not up to par in the "Data Collection" section, making it difficult to follow. Please revise.*

We have revised the “Data Collection” section by adding more details to make it easier to understand. Some sentences were also deleted or rephrased.

*4. The described algorithm for identifying UC patients seems reasonable, though validity is not mentioned. Has this algorithm been tested against e.g. chart data, and with what results?*

The algorithm is originated from a previous Hungarian study by Kurti et al. (doi: 10.1016/j.dld.2016.07.012). We lacked the necessary data to validate this algorithm ourselves.

*5. Though I assume this to be the case, it should be mentioned that also the matched controls were also assessed for CRC using the described method for the UC patients (the cases).*

Unfortunately, no other data (comorbidities, etc.) except the date of birth, gender and the date of death could be obtained for the general population in this study; therefore, further analysis of the matched controls couldn’t be performed.

We have clarified this circumstance in the Data collection and Discussion parts of the manuscript.

*6. In the statistics section, there is no mention of missing data and how this was handled. Also, was the Cox model tested for assumptions?*

The Statistical analysis section was improved by inserting the following information:

Due to the claims nature of the data, missing data are undiscoverable in most cases. If a certain intervention or diagnosis was not recorded, there is no chance that it could be imputed in any way. Therefore, no handling of missing data was performed.

The proportional hazards assumption for the Cox model was tested using plots of Schoenfeld residuals.

*7. The authors state that the decrease seen in UC incidence is a function of their diagnosis capture. This should be stated in the discussion, and how sure are the authors of this fact? Moreover, please analyze this statistically also (p value for difference/trend).*

Due to the unreliability of the results obtained, we have decided to reduce the information presented. Fig 2. was removed altogether and only a point prevalence for the year 2015 was presented. As this is explained in the results section, we did not see the need to repeat it in the discussion section.

*8. It would be interesting to see the incidence of CRC in the matched population also.*

Unfortunately, no other data (comorbidities, etc.) except the date of birth, gender and the date of death could be obtained for the general population in this study; therefore, further analysis of the matched controls couldn’t be performed.

We have clarified this circumstance in the Data collection and Discussion parts of the manuscript.

*9. State p value for difference when comparing median survival in CRC UC versus non-CRC UC.*

Due to the obvious differences in the survival of CRC patients and all patients (5-year survival rates of 65% vs 86%), this formal analysis was not performed.

*10. Survival curves should have Life tables and log rank tests, perhaps also confidence intervals.*

Life tables were added to the corresponding figures. The p-value for log-rank tests was also included to figures with comparisons. Shading was used to display the confidence intervals for the survival curves.

*11. There is a paucity of other population-based studies (apart from Hungarian ones) in the discussion. E.g. Rutegard 2016 Scand J Surg found 9% CRC incidence after 38 years of screening colonoscopy in a defined UC cohort and should be cited, along with the St Marks study (Rutter 2006 Gastroenterology). Is there any screening programmes for UC in Hungary?*

We revised the discussion section and added the mentioned population-based studies.

The CRC-screening program has been started in 2019, before that, some pilot studies were conducted. No special UC-CRC screening program is available yet in Hungary, but we follow the ECCO-guidelines on outpatient follow-up visits.
